# Dissection of the Genetic Architecture of Rice Tillering using a Genome-wide Association Study

**DOI:** 10.1186/s12284-019-0302-1

**Published:** 2019-06-20

**Authors:** Su Jiang, Dan Wang, Shuangyong Yan, Shiming Liu, Bin Liu, Houxiang Kang, Guo-Liang Wang

**Affiliations:** 1 College of Agronomy, Hunan Agricultural University, Changsha, 410128 China; 2 State Key Laboratory for Biology of Plant Diseases and Insect Pest, Institute of Plant Protection, Chinese Academy of Agricultural Sciences, Beijing, 100193 China; 3 Tian Jin Key Laboratory of crop genetic breeding, Tianjin Crop Research Institute, Tianjin Academy of Agriculture Sciences, Tianjin, 300112 China; 4 Guangdong Key Laboratory of New Technology in Rice Breeding, Rice Research Institute, Guangdong Academy of Agricultural Sciences, Guangzhou, 510640 China; 5 Department of Plant Pathology, Ohio State University, Columbus, OH 43210 USA

**Keywords:** Rice, Tiller number, Genome wide association study, Genetic architecture, Single nucleotide polymorphism, Linkage disequilibrium, Gene expression

## Abstract

**Background:**

Rice tiller number (TN) is one of the most important components associated with rice grain yield. Around one hundred rice TN genes have been identified, but dissecting the genetic architecture of rice TN variations remains difficult because of its complex trait and control by both major genes and quantitative trait loci (QTLs).

**Results:**

In this study, we used a subset of the rice diversity population II (S-RDP-II), genotyped with 700,000 single nucleotide polymorphisms (SNPs), to identify the loci associated with tiller number variations (LATNs) through a genome-wide association study (GWAS). The analysis revealed that 23 LATNs are significantly associated with TN variations. Among the 23 LATNs, eight are co-localized with previously cloned TN genes, and the remaining 15 LATNs are novel. DNA sequence analysis of the 15 novel LATNs led to the identification of five candidate genes using the accessions with extreme TN phenotypes. Genetic variations in two of the genes are mainly located in the promoter regions. qRT-PCR analysis showed that the expression levels of these two genes are also closely associated with TN variations.

**Conclusions:**

We identified 15 novel LATNs that contribute significantly to the genetic variation of rice TN. Of these 15, the five identified TN-associated candidate genes will enhance our understanding of rice tillering and can be used as molecular markers for improving rice yield.

**Electronic supplementary material:**

The online version of this article (10.1186/s12284-019-0302-1) contains supplementary material, which is available to authorized users.

## Background

Rice (*Oryzae sativa* L.) is one of the main cereal crops that feeds more than half of world’s population (Khush 2005). Tiller number (TN) and panicle morphology are two key factors associated with rice grain yields (Wang & Li 2011). Rice TN is also used as a model system for studying the branching mechanisms in monocotyledonous plants (Li et al. 2003). Two developmental steps are used for regulating shoot branches: the first is the formation of axillary meristem, and the second is the growth of axillary buds (Yasuno et al. 2007). For a particular variety of rice, its tillering capacity is largely determined by genetic factors and can be affected by environmental conditions, such as light, temperature, plant density, nutrients and water supply (Wang & Li 2005).

Three important monoculm genes have been identified thus far in rice: *MOC1*, *MOC2*, and *MOC3*. *MOC1* (*Os06g40780*) on chromosome 6 is a member of the GRAS (GAI, RGA and SCR) family of proteins that promotes axillary bud outgrowth. In the *moc1* mutant, a 1.9-kb retrotransposon inserted in *MOC1* interrupts the gene and leads to a single main culm phenotype (Li et al. 2003). *MOC2* (*Os01g64660*) on chromosome 1 encodes a fructose-1,6-bisphosphatase (FBP1), which is imperative for tiller bud outgrowth in rice. A Tos17 transposable element inserted into exon 4 of *MOC2* results in significantly reduced TN when compared to wild-type rice (Koumoto et al. 2013). *MOC3* (*Os04g56780*)/*TAB1*/*OsWUS* on chromosome 4 is requird for axillary buds formation (Lu 2015b). Some of the tillering dwarf genes that have been identified are also associated with effective TN by way of the strigolactone signaling pathway. For example, *D3 (Os06g06050)* on chromosome 6 encodes an F-box leucine-rich repeat (LRR) protein, which controls the axillary bud activity (Shinji et al. 2005). *d27* (*Os11g0587000*, IRGSP-1.0) on chromosome 11 encodes an iron-containing protein, which regulates rice tiller bud outgrowth (Lin et al. 2009). *HTD2/D88/D14* (*Os03g10620*) on chromosome 3 encodes esterase/lipase/thioesterase, which negatively regulates tiller bud outgrowth (Liu et al. 2009). *D53* (*Os11g01330*) on chromosome 11 is a repressor of strigolactone signaling (Jiang et al. 2013). Genes in the plant hormone pathway are also involved in TN regulation, including the cytokinin oxidase/dehydrogenase gene *OsCKX2* (*Os01g10110*) (Yeh et al. 2015), the caroteniod isomerase gene *MIT3* (*Os11g36440*) (Liu et al. 2017) and the putative auxin efflux carrier *OsPIN1* (*Os02g50960*) (Xu et al. 2005). Furthermore, the amino acid transporter *OsAAP3* (*Os06g36180*) negatively regulates bud outgrowth and rice TNs (Lu et al. 2018), and heme activator protein gene *OsHAP2E* (*Os03g29760*) increases photosynthesis and TNs (Alam et al. 2015).

Rapid development of both next-generation genome sequencing and genotyping technologies supports construction of high-density SNP maps for a population that is both technically feasible and cost effective. The genome-wide association study (GWAS) method based on the high-density SNP maps and large diverse germplasm collections has become a new strategy for efficient and high-throughput gene identification in multiple species (Liu & Yan 2019). The first GWAS on 14 agronomic traits identified three loci associated with the TN (Huang et al. 2010). Another GWAS led to the identification of a TN-associated 200 kb size locus that included 32 canidate genes (Lu et al., 2015c). Dozens of loci associated with both TN variation (Wu et al. 2018) and tiller angle-variation (Dong et al. 2016) were recently identified using different natural rice populations. Because TN is a complex trait and the genetic basis of tillering is not completely understood, more research is needed to fully understand the genetic architecture of rice TN variations and identify more loci associated with tiller numbers (LATNs) in diverse germplasms.

In this study, we investigated the rice TN using a subset of the rice diversity population II (S-RDP-II) (McCouch et al. 2016). We identified 23 LATNs that were significantly associated with TN variations. Among those 23 loci, 12 cloned TN-related genes are co-localized within the 8 LATN regions, and the remaining 15 loci are novel. Further genetic sequence comparison and gene expression analyses enabled us to identfy 5 novel TN-associated genes in the 15 novel LATNs. Our study revealed that, besides genetic sequences, gene expression levels are also important for rice TN variations. The novel LATNs identified in this study could be useful for further dissection of the complex genetic architecture of rice TN variations and molecular breeding of high yielding rice cultivars.

## Methods

### Plant materials

The rice diversity population and the high diversity SNP maps (700,000 SNPs) are publicly available (McCouch et al. 2016; http://www.ricediversity.org/data/).

### Field experiments

All rice seeds were soaked in clean water, at 37 °C for 36 h, replaced with clean water every 9 h, then germinated for 16 h. The seeds were sowed in Wuqing, Hebei province on May 1, 2018, and the 25-day-old seedlings were transplanted into the field. Transplanting density was 16 cm × 20 cm with three rows and eight seedlings. A complete randomized block design was used with three replicates for each accession. The three plants in the middle were investigated for the TN at the later tillering stage. The mean TN from three replicates was used for the GWAS.

### The GWAS method of the S-RDP-II population

The GWAS method was similar to that previously described in our study (Kang et al. 2016), except using a new version of the Tassel program (Tassel 5.0). Briefly, the mixed linear model (MLM) was selected in which the genetic marker-based kinship matrix (K), generated by Tassel with the SNP dataset, was used jointly with population structure (Q). We selected candidate LATNs using the following standard: a genomic region with ≤200 kb with at least two significant associated SNPs (*p*-value threshold ≤1E-4).

### Candidate gene sequencing and sequence analysis

The sequences of the five candidate genes were downloaded from the Rice Genome Annotation Project website (http://rice.plantbiology.msu.edu/analyses_search_blast.shtml). The full-length sequence of the candidate genes contains from ~ 1500 bp upstream of 5’UTR to 500 bp downstream of 3’UTR. The Gene specific primers were designed for cloning those genes in the accessions which have extreme high TN and extreme low TN. DNA sequencing of the cloned genes was finished by the TSINGKE Company in Beijing. Sequence alignment was performed with DNAMAN. All primers used in the study are shown in Additional file 1: Table S1.

### RNA extraction and candidate gene expression analysis

Rice leaf samples were collected from rice cultivars in the paddy field. RNA and DNA extraction were performed with Trizol reagent (Invitrogen) and CTAB buffer, respectively. cDNA synthesis was performed according to the manufacturer’s instructions for the cDNA synthesis kit (Vazyme). qRT-PCR was performed on ABI Prism 7500 PCR instrument with 10 μl 2 × SYBR Green Mix (GeneStar), 5 μl cDNA, 0.5 μl gene-specific primers and 4 μl filter water. Rice ubiquitin gene was used as an internal control for normalization. The qRT-PCR primers are listed in Additional file 1: Table S1.

## Results

### TN variations in the S-RDP-II population

The rice TN phenotype is associated not only with genetic elements but also with environmental conditions, such as light and nutrition (Wang & Li 2005). Temperate Japonica (TEJ) rice belongs to the northern ecotype in China, and subtropical indica (IND) rice belongs to the southern ecotype. To investigate whether the growth conditions in the two regions affect rice tillering, we selected 10 rice accessions from the previously studied rice diversity population 1 (RDP1). Among the 10 accessions, five had low TN and five had high TN based on their phenotypes in our Langfang greenhouses in Beijing. We grew these 10 accessions in the field of both North China (Beijing) and South China (Changsha). Results showed that the TN of all the cultivars were similar between North China (Additional file 2: Figure S1) and South China (Additional file 2: Figure S2), indicating that hereditary factors, rather than growth conditions, are most crucial for rice TN variations.

**Fig. 1:**
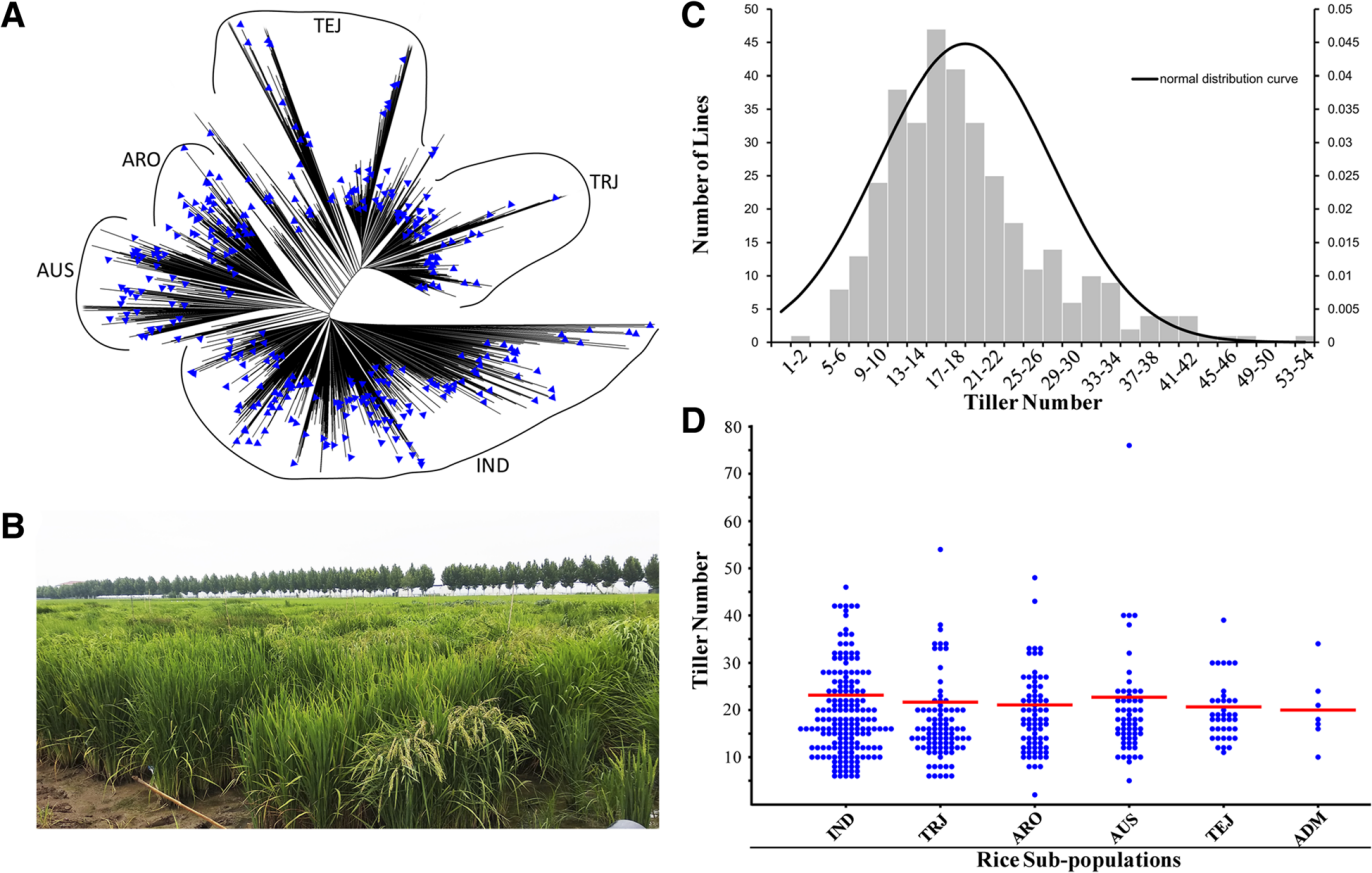
The structure of the S-RDP-II population and its TN variation in the field. **a** Phylogenetic tree of the RDP-II population in which the 350 accessions (S-RDP-II) are indicated by blue triangles. Six subpopulations are IND, TEJ, TRJ, AUS, ARO and ADM. **b** Photo of S-RDP-II cultivars in the field when TN was counted. **c** The TN variation distribution in the S-RDP-II population. X-axis represents TNs and Y-axis represents the number of accessions with the TN interval. **d** The TN variation in the subpopulations. Each dot represents a rice accession in the subpopulation. Red line represents the average value of TNs in the six subpopulations

To dissect the genetic structure of rice tillering, we selected 350 accessions based on two principles from the RDP-II that contained about 1500 accessions. First, we made a phylogenetic tree using the whole RDP-II population and manually checked the branches of the tree to make sure no closely related accessions were selected. We then increased the seeds of the RDP-II population in the field with subtropical climate and selected those with normal growth and enough seeds. The population of the selected 350 accessions (S-RDP-II) contained six sub-populations, including IND, TEJ, tropical japonica (TRJ), aromatic (ARO), aus (AUS) and admixture (ADM) with 145, 31, 72, 42, 53 and 7 accessions, respectively (Fig. 1a). We calculated the TN of S-RDP-II at the mature stage (Fig. 1b). The average TN of the population is 19.0, with a minimum of 5 (accession: 121017) and maximum of 76 (accession: 117434) tillers at the mature stage (Additional file 1: Table S2). The distribution of the TN in the population was nearly a normal distribution (Fig. 1c). About 52.3% accessions (183 of 350) had 12–20 tillers. The average TN among the different sub-populations was similar, i.e., 19.0 (± 8.8SD [standard deviation]), 20.0 (± 6.3SD), 17.5 (± 8.2SD), 19.2 (± 8.8SD), 20.5 (± 11.6 SD) and 20.0 (± 7.0SD) in IND, TEJ, TRJ, ARO, AUS and ADM, respectively (Fig. 1d). The relatively big SD value in each subpopulation indicated that the TN variation is large within the subpopulations.

### Identification of LATNs in the rice genome

**Fig. 2:**
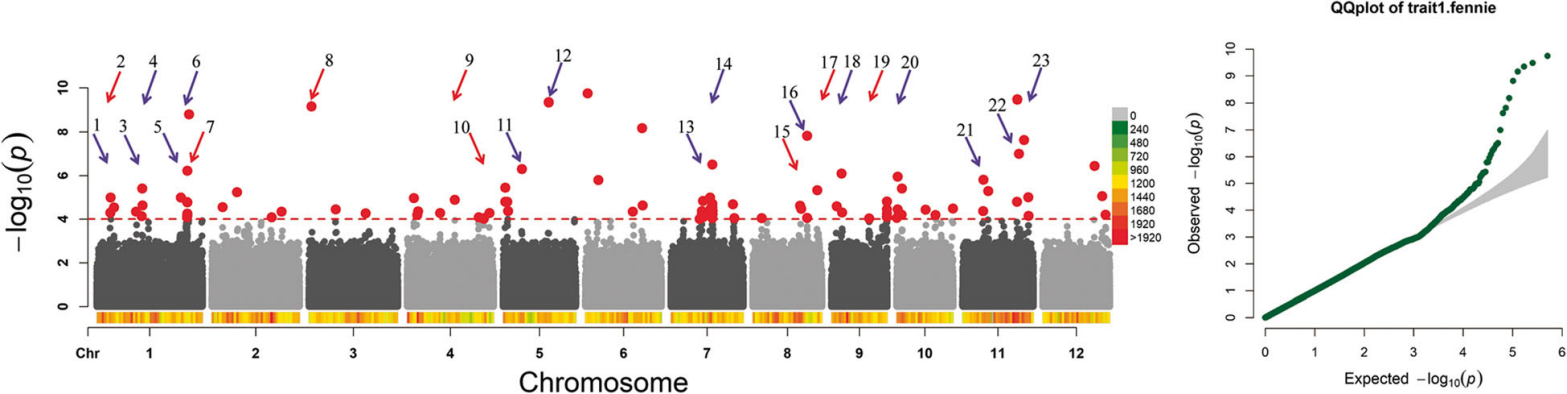
GWAS of the TN variation using the S-RDP-II population and 700,000 SNP maps. X-axis represents 12 rice chromosomes and Y-axis represents the converted *P-value* (−log_10_(*P*). Each red dot represents one SNP. Arrows represent the 23 LATNs: red arrows are LATNs co-localized with previously cloned TN associated genes, and blue arrows are the novel loci. Panel on the right is the correlated QQ plots figure

Using the TN phenotypes obtained from the field in this study (Additional file 1: Table S2) and the 700,000 SNP maps of the S-RDP-II, we performed the GWAS and identified 23 LATNs (Table 1). The 23 loci were distributed onto all of the rice chromosomes except for 2, 8 and 9 (Fig. 2). A simple additive model in the Statistical Analysis System (SAS) was used to determine whether the genotypes of the 23 LATNs were correlated with the TN phenotypes. The 15 LATNs with the most significantly associated SNPs from the 23 LATNs were included in the model. The correlation analysis showed that the 15 LATNs explained 73.4% of the TN variation in the S-RDP-II population. The 15 loci are LATN-1, 2, 3, 6, 8, 9, 10, 11, 13, 15, 17, 18, 21, 22 and 23 (Table 1). These results indicate that multiple loci control the complex genetic architecture of the TN variation in rice.

**Table 1:** The 23 LATNs and their rice genomic information

LATN	Name_of_Top-SNPs	SNP_Types	Chr.	Positions	*p*-value	Annotation	Reference
1	SNP-1.5635230.	A/G	1	5,636,231	1.04E-05	Novel_1	/
2	SNP-1.7037988.	C/G	1	7,038,989	2.95E-05	*CCP1*	*(*Yan et al., 2015*)*
3	SNP-1.16070366.	A/G	1	16,071,393	4.56E-05	Novel_2	/
4	SNP-1.18536011.	A/G	1	18,537,058	4.03E-06	Novel_3	/
5	SNP-1.36879662.	T/C	1	36,880,706	1.68E-05	Novel_4	/
6	SNP-1.37003809.	C/G	1	37,004,853	6.07E-07	Novel_5	/
7	SNP-1.37664127.	A/G	1	37,665,171	1.56E-09	*REL1, MOC2, PLA2*	*(*Chen et al., 2015*),(*Koumoto et al., 2013*),(*Kawakatsu et al., 2006*)*
8	SNP-3.1121412.	T/C	3	1,122,415	6.93E-10	*OsDCL1*	*(*Zhang et al., 2015*)*
9	SNP-4.19405464.	C/G	4	19,577,430	1.31E-05	*OsAFB2,LAX2*	*(*Xia et al., 2012*), (*Tabuchi et al., 2011*)*
10	SNP-4.33497579.	T/G	4	33,682,697	5.26E-05	*MOC3, OsGS2*	*(*Lu et al., 2015b*), (*Cai & Xiao, 2010*)*
11	SNP-5.7728359.	C/T	5	7,728,419	5.05E-07	Novel_6	/
12	SNP-5.18643585.	C/T	5	18,706,103	4.52E-10	Novel_7	/
13	SNP-7.12702296.	A/G	7	12,703,290	4.29E-05	Novel_8	/
14	SNP-7.16935997.	A/T	7	16,936,991	3.16E-07	Novel_9	/
15	SNP-8.19480736.	A/G	8	19,483,450	2.72E-05	*PAY1*	*(*Zhao et al., 2015*)*
16	SNP-8.22337472.	A/G	8	22,340,186	1.53E-08	Novel_10	/
17	SNP-8.26426233.	T/C	8	26,428,948	4.74E-06	*OsPIN5b*	*(*Lu et al., 2015a*)*
18	SNP-9.4181935.	T/C	9	4,182,936	8.20E-07	Novel_11	/
19	SNP-9.22712391.	A/T	9	22,712,873	5.89E-05	*OsAHP2*	*(*Sun et al., 2014*)*
20	SNP-10.504597.	A/G	10	505,622	1.13E-06	Novel_12	/
21	SNP-11.8537869.	A/C	11	8,543,260	1.60E-06	Novel_13	/
22	SNP-11.21947025.	A/C	11	22,413,155	3.33E-10	Novel_14	/
23	SNP-11.26432763.	T/C	11	26,904,377	1.02E-05	Novel_15	/

When compared with 90 previously cloned TN associated genes (Additional file 1: Table S3), we found that 12 cloned genes were co-localized with 8 LATNs including LATN-2, 7, 8, 9, 10, 15, 17 and 19 and the other 15 loci were novel loci. Detailed information of the known genes is listed in Table 1. It is most notable that *MOC3* is co-localized with LATN-10. A previous study showed that the single tiller mutant *moc3* is caused by one SNP mutation (Lu et al., 2015b); we subsequently investigated whether the association at LATN-10 was due to *MOC3*’s sequence polymorphism or not. We selected 5 accessions with few TN and 6 accessions with more TN that contained the corresponding genotype at the locus. Sequencing and sequence alignment of the full length fragment (including promoter and coding region) of *MOC3* identified 11 allelic variations: 10 SNPs and one 12-bp InDel (Additional file 1: Table S4), however, none of those variations was the same as the functional mutation in *moc3* reported previously (Lu et al., 2015b). All of these variations were associated with the TN phenotypes. It is interesting to mention that all of these variations were located in the putative promoter region, indicating that the variations may be associated with *MOC3*’s expression rather than its gene coding sequences.

### Gene ontology analysis of the 15 novel LATNs

We analyzed the 200 kb genomic sequences (100 kb flanking the left and right side of the most significantly associated SNPs) of the 15 novel LATNs from the reference genome of the japonica cultivar Nipponbare (NPB). These 15 loci encoded a total of 435 genes (Additional file 1: Table S5). Among these genes, 142, 98 and 36 genes were annotated as unknown proteins, retrotransposon and transposon proteins, respectively. The other 159 genes were annotated with putative functions using the gene annotation tool (http://www.ebi.ac.uk/interpro/search/sequence-search). About 55% (88 of 159) of the gene products belong to six classes: 39 enzyme proteins, 12 chloroplast and photosystem related proteins, 10 protein kinases, 10 NBS-type proteins, six NADPH-dependent oxidoreductase, four transcription factors, four plant hormone related proteins and three cytochrome P450.

### Five novel genes associated with TN variations

To further validate the association between the candidate genes and the TN phenotypes, we selected seven genes (Additional file 1: Table S6) either in the center of the peak value region of the associated SNPs or annotated as plant hormone-related genes from the 15 novel LATNs for sequencing and quantitative real time polymerase chain reaction (qRT-PCR) analysis. We designed the specific primers for each candidate gene (Additional file 1: Table S1) and amplified all of the genes, except *Os05g14010* at LATN-11 and *Os11g37840* at LATN-22, which could not be amplified by their specific primers in most of the tested rice accessions. The five genes were *Os01g28690* at LATN-3, *Os05g32120* at LATN-12, *Os07g28890* at LATN-14 and *Os11g15130* and *Os11g15210* at LATN-21. The genomic sequencing results confirmed that all five of the genes contained SNPs which were associated with the TN variation. qRT-PCR using the extreme low and high TN varieties and “two sample t-test for means” analysis indicated that the expression levels of the genes *Os01g28690* and *Os05g32120* were also closely correlated with the TN variation.

**Fig. 3:**
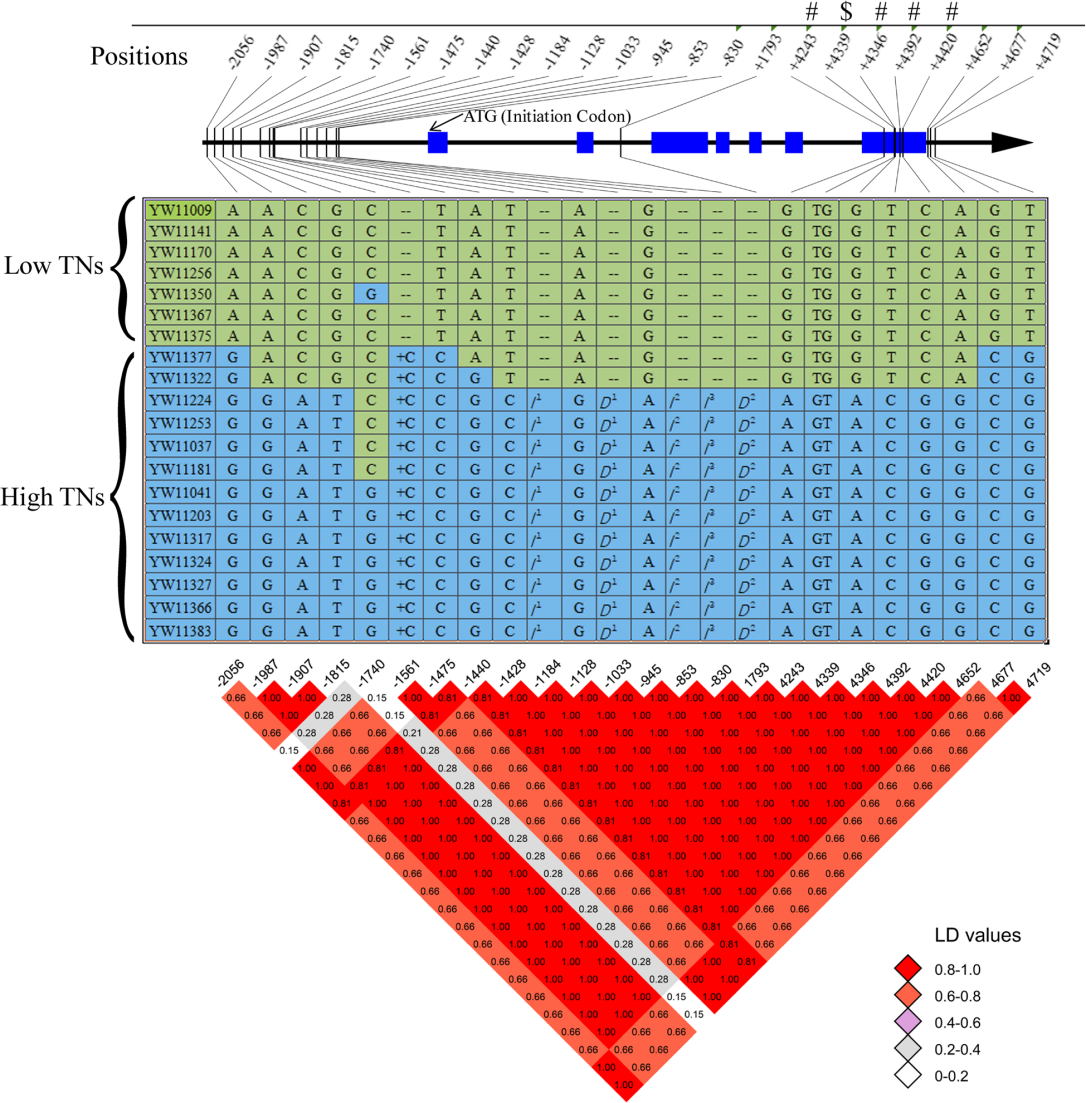
Sequence analysis of the candidate gene *Os01g28690* in the rice accessions with extremely low and high TNs. Tilted numbers at the top represent the position related to the initiation codon of ‘ATG’; ‘-’ indicates upstream of ‘ATG’; ‘+’ indicates downstream of ‘ATG’; “#” is a synonymous mutation; “$” indicates a missense mutation from tryptophan (W) to Valine (V). Arrow shows the direction of *Os01g28690*; blue rectangles indicate the gene exon regions. The middle panel is the detail sequence variation among the extreme low and high TN accessions. ‘A’, ‘T’, ‘G’ and ‘C’ in the table are the bases. ‘--’ indicates that the sequence in the accessions is the same as the reference sequence, and “+C” indicates insertion of one base pare. ‘*I*^1^’, ‘*I*^2^’ and ‘*I*^3^’ represent insertion of ‘ATTACTAG’, ‘CTCGCGGA’ and ‘CCTTCG’, respectively, while ‘*D*^1^’ and ‘*D*^2^’ represent deletion of ‘ATGTTATCAATTTGACAACATGGC’ and ‘ATCT’, respectively. The bottom panel is the LD-decay of the TN variation in the region. The LD values are included in the blocks with different colors

*Os01g28690* encodes a nucleoporin autopeptidase domain-containing protein located in the center of LATN-3. We selected seven low TN varieties and 13 high TN varieties, which were correlated with the genotypes at LATN-3. We identified 24 allelic variations in both the promoter and gene coding regions. Fifteen, six and three of the allelic variations were located in the putative promoter, coding and UTR regions (Fig. 3), respectively. Five variations, including one bp Indel and four SNPs, were 100% associated with the high/low TNs. Among the five top associated variations, three were located in the putative promoter region (bases 2076, 1561 and 1475 before the initiation codon) and two were located in the putative UTR regions (positions 4677 and 4719, representing bases 42 and 84 after the stop codon, respectively). It is most notable that five allelic variations were located in the seventh exon of *Os01g28690*, four of which cause synonymous mutations (Fig. 3). However, one mutation (TG/GT), located on 4339 after the ‘ATG’ initiation codon, is a missense mutation, leading to the substitution of aa from tryptophan (W) coding by ‘TGG’ to valine (V) coding by ‘GTG’.

*Os05g32120* encodes an alpha 1,3-xylosyltransferase, located in the center of LATN-12. Using six low TN varieties and seven high TN varieties, which are correlated with the genotypes at LATN-12, we identified 7 allelic variations from sequencing the full-length gene fragment (~ 2 kb of the promoter and coding region, and ~ 1 kb after the stop codon). Six of these variations were located in the putative promoter region (positions 1154, 1087, 1034, 1029, 911 and 832 before the initiation codon), and one was located in the second intron. Three allelic variations were 100% associated with the TN variation (Additional file 2: Figure S3).

**Fig. 4:**
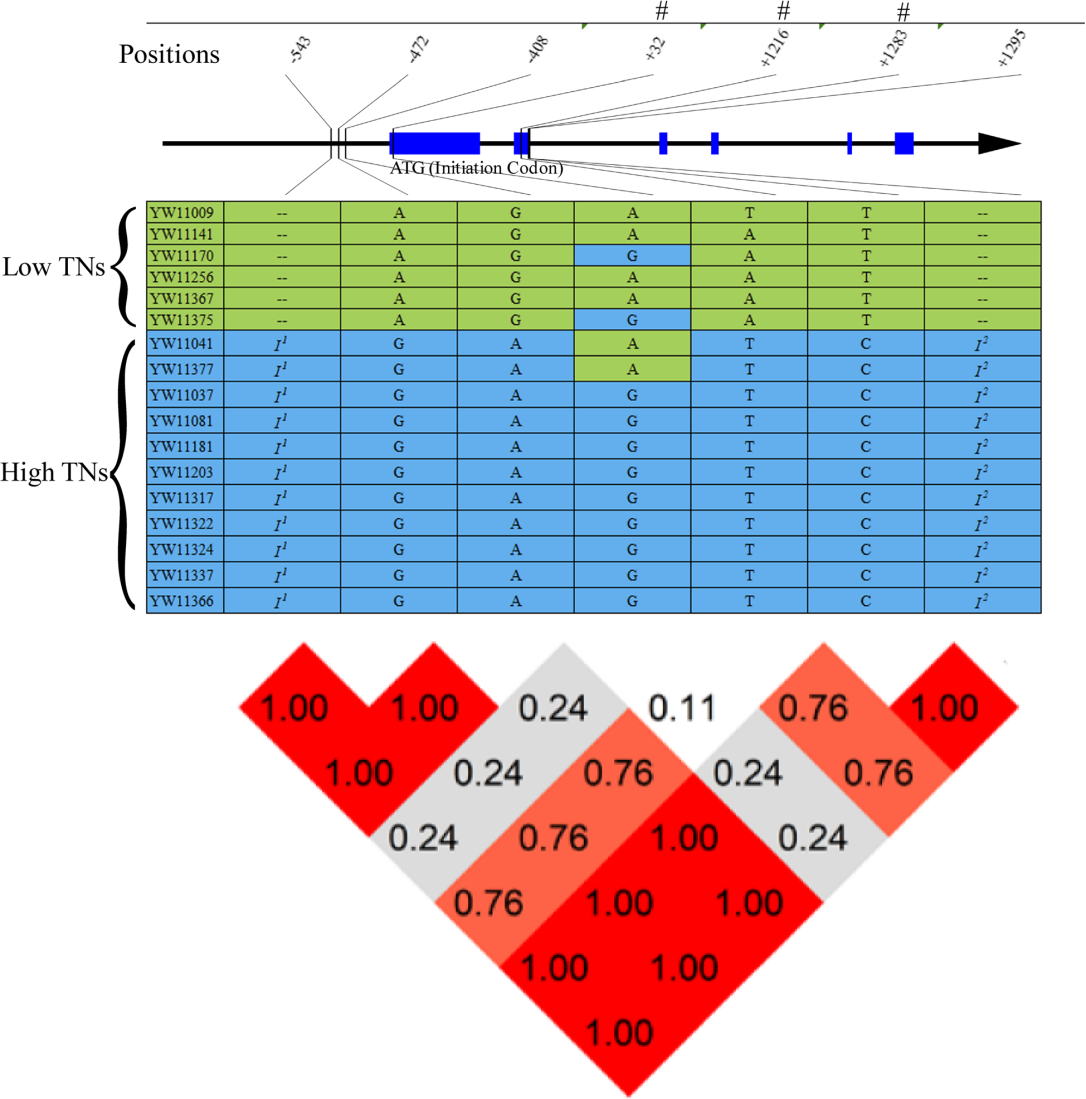
Sequence analysis of the candidate gene *Os07g28890* in the rice accessions with extremely low and high TNs. Tilted numbers at the top indicate the position related to the initiation codon of ‘ATG’; ‘-’ indicates upstream of ‘ATG’; ‘+’ indicates downstream of ‘ATG’; “#” is a synonymous mutation. The middle panel shows the sequence variation in the extreme low and high TN accessions. . ‘A’, ‘T’, ‘G’ and ‘C’ in the table are the four bases. ‘--’ indicates that the sequences are the same as the reference genome. ‘*I*^1^’ and ‘*I*^2^’ represent insertion of ‘AGCTAGCTAGCT’ and ‘ATGCATCCATATATCATGATCTAGCAGGTATCATTATACTCTACATATTGTCAATTTTTTTCAGAATTTTTCACAACTATTTGCATCGAATTTGGAAGAAAAAGGTATACTAGGGGATATCCCCTCGAGGGATTAGAATCCACTCCCTTTCTAAATTAATGTACTATTTGATTATTGTTTTATTCAAAAA’ mutations, respectively. The bottom panel is the LD-decay of the TN variation in the region. The LD values are included in the blocks with different colors

*Os07g28890* encodes an ethylene-responsive protein, which is located in the center of LATN-14. From the gene sequences of 17 TN varieties (6 low and 11 high TN), we identified seven allelic variations. Unlike other candidate genes, only three of the variations were located in the putative promoter region (positions 543, 472 and 408 before the initiation codon), and four variations were located within the gene. The first one was located in the first exon (G-to-A mutation) and caused a missense mutation from glycine (G) to glutamic acid (E). The second one was a synonymous mutation located in the second exon (A-to-T mutation) (Fig. 4). The remaining two were located in the second intron region: one very close to the GT--AG splicing site (GTAT……AG, the fourth base ‘T’ mutation to ‘C’), and the other a 220 bp deletion in the low TN varieties (or a 220 bp insertion in the high TN varieties) close to the start of the splicing cite in the second intron (Fig. 4). We speculate that the mutations may lead to alternative splicing and functional changes in *Os07g28890*.

**Fig. 5:**
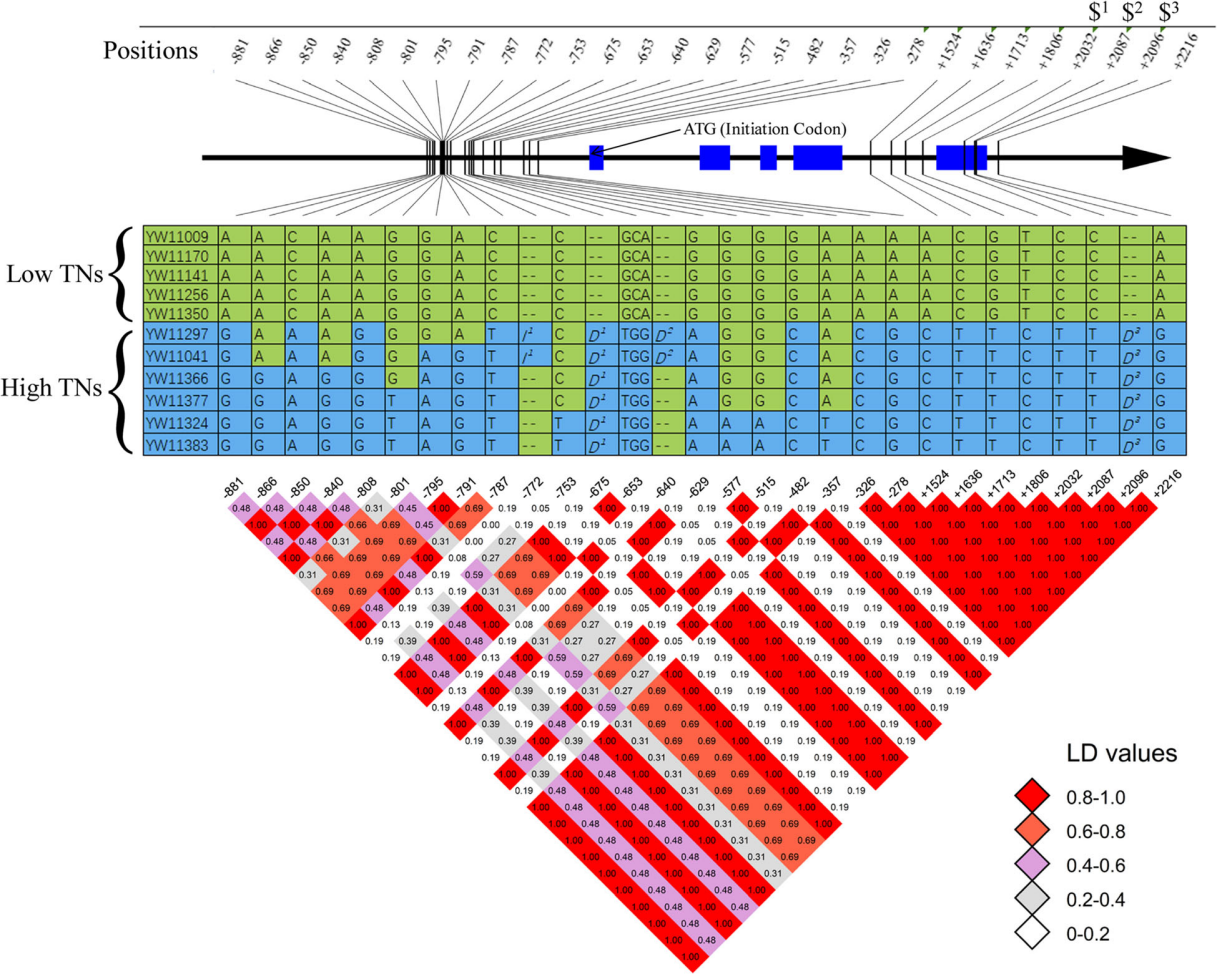
Sequence analysis of the candidate *Os11g15130* gene in the rice accessions with extremely low and high TNs. Tilted number at the top represents the position related to the initiation codon of ‘ATG’; ‘-’ and ‘+’ indicate the upstream and downstream of ‘ATG’, respectively. ‘$^1^’ indicates a missense mutation from P to S. ‘$^2^’ indicates a missense mutation from Alanine (A) to Valine (V), while ‘$^3^’ indicates a frameshift mutation caused by the deletion of ‘AATT’. Arrow shows the direction of *Os11g15130*; blue rectangles indicate the gene exon regions. The middle panel is the sequence variations among the extremely low and high TN accessions. The sequence identical regions are not shown in the table due to space limitation. “ATGC” indicates the four bases. ‘--’ indicates that the sequences are the same as the reference genome. ‘*I*^1^’ represents an insertion of ‘TAACT’. ‘*D*^1^’, ‘*D*^2^’ and ‘*D*^3^’ represent deletions of ‘TGCTAGCTGC’, ‘AC’ and ‘AATT’, respectively. The bottom panel is the LD-decay of the TN variation in the region. The LD values are included in the blocks with different colors

*Os11g15130* and *Os11g15210* are located in the middle of LATN-21. *Os11g15130* encodes a jasmonate O-methyltransferase, and *Os11g15210* encodes an anthocyanin regulatory protein. We analyzed the DNA sequences of 21 cultivars and identified that more than half (16 of 29) of the allelic variations of *Os11g15130* were closely associated with TN variations. Three of the allelic variations were located in the last exon: one (base 2032 after the initiation codon, T/C SNP) lead from the proline (P) to serine (S) mutation; the second (base 2087 after the initiation codon region, T/C SNP) lead from the Alanine (A) to Valine (V) missense mutations; and the third (base 2096 after the initiation codon region, ‘AATT’ Indel) resulted in a frameshift mutation (Fig. 5). For *Os11g15210*, we identified very few allelic variations (3 of 26) that were tightly associated with TN variations. Only one of these mutations was located in the exon region and was a synonymous mutation (Additional file 2: Figure S4).

**Fig. 6:**
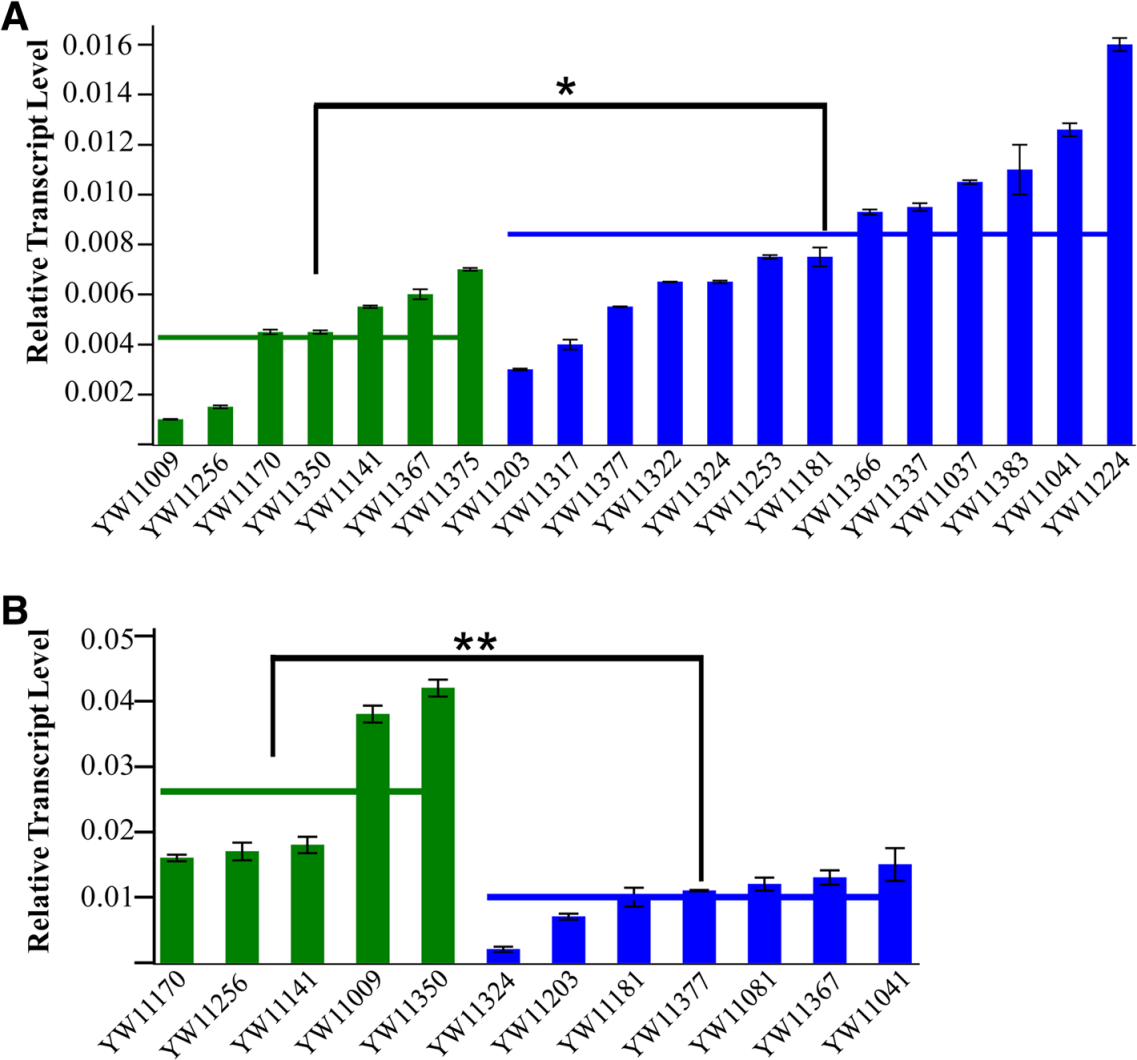
qRT-PCR analysis of *Os01g28690* (**a**) and *Os05g32120* (**b**) between the extremely low and high TN accessions. X-axis represents the different rice accessions. Green bars are the low TN accession, and blue bars are the high TN accessions. Y-axis represents the relative gene transcript levels. The green and blue horizontal lines are the average gene expression levels in the low and high TN rice accessions, respectively. Black bars indicate the difference between the means of TNs in low and high TN accessions.’*’ marks significant differences (*P* = 0.0132), while ‘**’ marks very significant differences (*P* = 0.0098)

As mentioned above, more than half of the allelic variations were closely associated with the TN variation and were located in the promoter/intron/UTR regions, rather than in the gene exon regions. We speculate that these variations may be associated with the expression levels of candidate genes. To test this speculation, we designed the gene specific primers (Additional file 1: Table S1) for qRT-PCR analysis of the five genes in the extreme low and high TN varieties. In addition, we performed the “two sample t-test for means” in the SAS system. The results indicated that the expression levels of two genes, *Os01g28690* and *Os05g32120*, were positively (*Os01g28690*, *P* = 0.0132) and negatively (*Os05g32120*, *P* = 0.0098) associated with TN variations (Fig. 6).

In summary, we demonstrated that five genes were closely associated with TN variations in the S-RPD-II population, and the TN phenotypes are closely associated with the expression levels of two genes. Further functional analysis of the identified genes will help us better understand the genetic architecture of rice TN variations.

## Discussion

Plant geneticists over a decade ago started to apply GWAS to dissect the genetic architecture of complex traits and map the loci that are associated with important agronomic traits (Feng et al. 2011). Numerous QTLs have been identified in more than 1000 GWAS projects that aimed to identify important loci in crops (Liu & Yan 2019). In this study, we identified 23 LATNs using a subset of the RPD2 population and GWAS. Among them, 12 known TN-related genes are co-localized within the 8 LATNs regions, and the other 15 loci are novel. These results confirmed that the GWAS method is effective for identifying the TN loci in rice. To evaluate the GWAS results, we selected five candidate genes in the LATN regions and confirmed their associations with the TN variation at both the genomic DNA and expression levels. Identification of these candidate genes has built a foundation for further detailed molecular analysis of their functions in rice tillering.

Previous studies showed that the genes involved in the phytohormone synthesis or degradation of strigolactone, auxin, cytokinin and carotenoid isomerase regulate or control the rice TN variation (Xu et al. 2005; Yeh et al. 2015; Liu et al. 2017). Ethylene is also known for regulating plant growth and development as well as regulating plant adaption to stressful conditions. Several important genes, such as ETO2 (Poupin et al. 2016), ERS1 (Deslauriers et al. 2015) and EIN2 (Guo et al. 2011), participate in regulating the ethylene signaling pathways in many plant growth processes. Additionally, jasmonate and its derivatives can regulate plant stress, growth and development (Wasternack 2007). In this study, we chose seven candidate genes, located either in the center of the peak value region or involved in the phytohormonal synthesis of the 15 novel LATNs, for further sequence and expression analyses. Two of the five candidate genes, *Os07g28890* and *Os11g15130,* encode an ethylene-responsive protein and a jasmonate O-methyltransferase protein, respectively. Knocking out and/or overexpressing of the two genes in transgenic rice will provide the information about their functions in rice tillering. Similar experiments can be performed for *Os01g28690* and *Os05g32120* because their expression levels are closely related to the TN variation.

## Conclusions

In this study, we identified 23 LATNs using S-RDP-II and GWAS. Twelve known TN-related genes are co-localized within the eight LATNs regions, and 15 are novel LATNs. We demonstrated that five novel candidate genes are closely associated with TN variations at the DNA level. Interestingly, the promoter sequence variation and their expression levels in two of the five genes are closely associated with the TN variation, indicating that both gene and expression variations are important for the genetic architecture of the rice TN. The five identified novel candidate genes in this study are good genetic materials for dissecting the genetic architecture of rice tillering.

## Additional files

Additional file 1: **Table S1.** Primers for PCR and qRT-PCR analysis of the candidate genes. **Table S2.** TN phenotypes of S-RDP-II population in the field. **Table S3.** List of the detailed gene information of the 90 cloned TN variation associated genes. **Table S4.** The haplotype analysis of the *Moc3* gene in the accessions with extreme low and high TN phenotypes. **Table S5.** The detailed information of the genes located in the 15 novel LATNs. **Table S6.** The seven candidate genes used for full length sequencing and expression analysis. (XLSX 50 kb)

Additional file 2: **Figure S1.** Field phenotypes of five low and five high TN accessions in Beijing, North of China. **Figure S2.** Field phenotypes of five low and five high TN accessions in Changsha, South of China. **Figure S3.** The haplotype analysis of the candidate gene *Os05g32120* in the accessions with extremely low and high TN phenotypes. **Figure S4.** The haplotype analysis of the candidate gene *Os11g15210* in the accessions with extremely low and high TN phenotypes. (DOCX 4965 kb)

## Data Availability

The datasets supporting the conclusions of this article are provided within the article and its additional files.
