# Supplementary material for: Dissection of the Genetic Architecture of Rice Tillering using a Genome-wide Association Study
Source: Rice (N Y). 2019 Jun 20;12:43. doi: 10.1186/s12284-019-0302-1 (PMC6586736; doi:10.1186/s12284-019-0302-1)
Supplement: Supplementary file 2 — Figure S1. Field phenotypes of five low and five high TN accessions in Beijing, North of China. Figure S2. Field phenotypes of five low and five high TN accessions in Changsha, South of China. Figure S3. The haplotype analysis of the candidate gene Os05g32120 in the accessions with extremely low and high TN phenotypes. Figure S4. The haplotype analysis of the candidate gene Os11g15210 in the accessions with extremely low and high TN phenotypes. (DOCX 4965 kb) [file 12284_2019_302_MOESM2_ESM.docx]

**Dissection of the genetic architecture of rice tiller number variation at the adult plant stage in the field using genome-wide association study**

Su Jiang, Dan Wang, Shuangyong Yan, Shiming Liu, Bin Liu, Houxiang Kang & Guo-Liang Wang


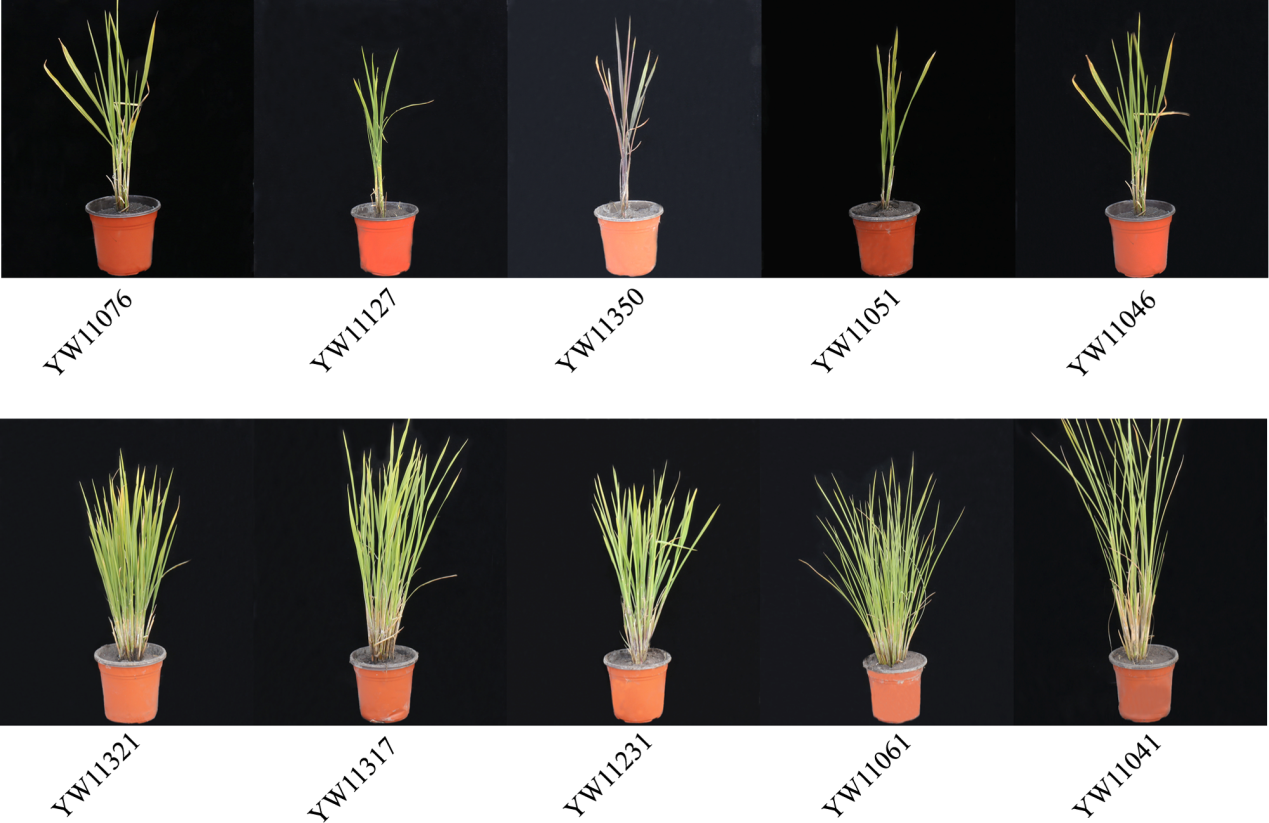


Figure S1. Field phenotypes of five low (upper panel) and five high (lower panel) TN rice accessions in Beijing, North of China.


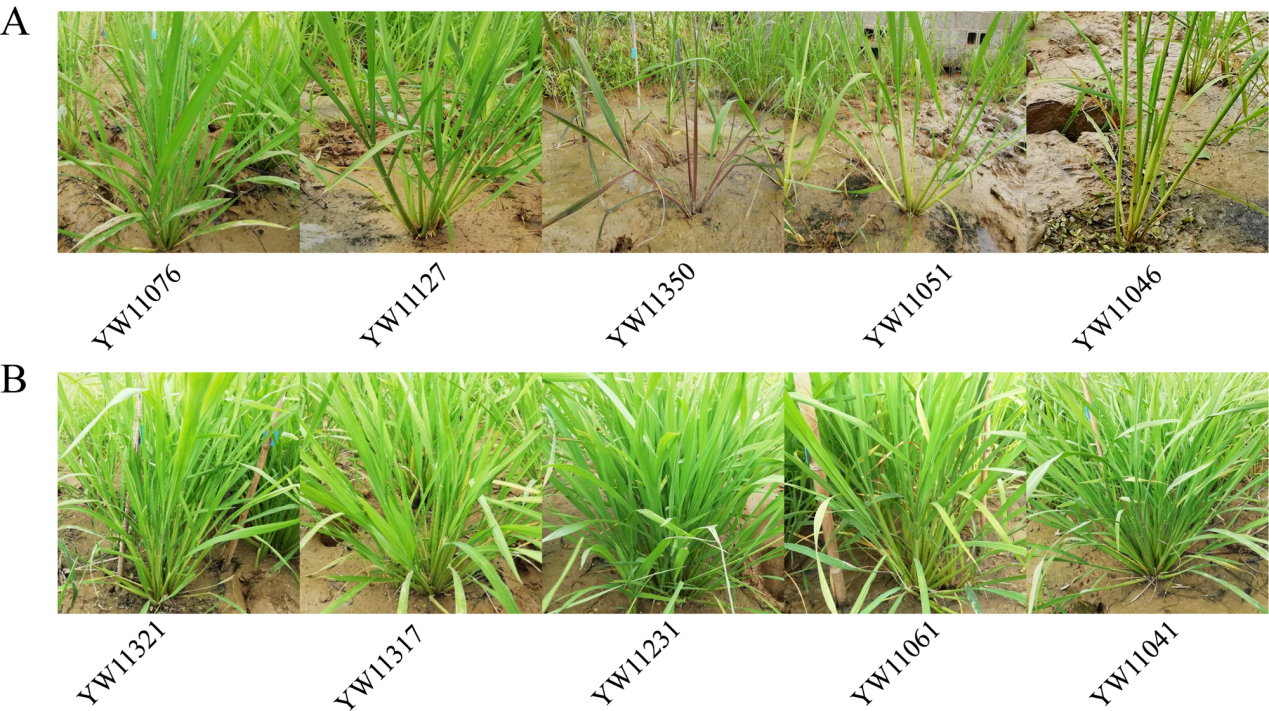


Figure S2. Field phenotypes of five low (panel **A**) and five high (panel **B**) TN rice accessions in Changsha, South of China.


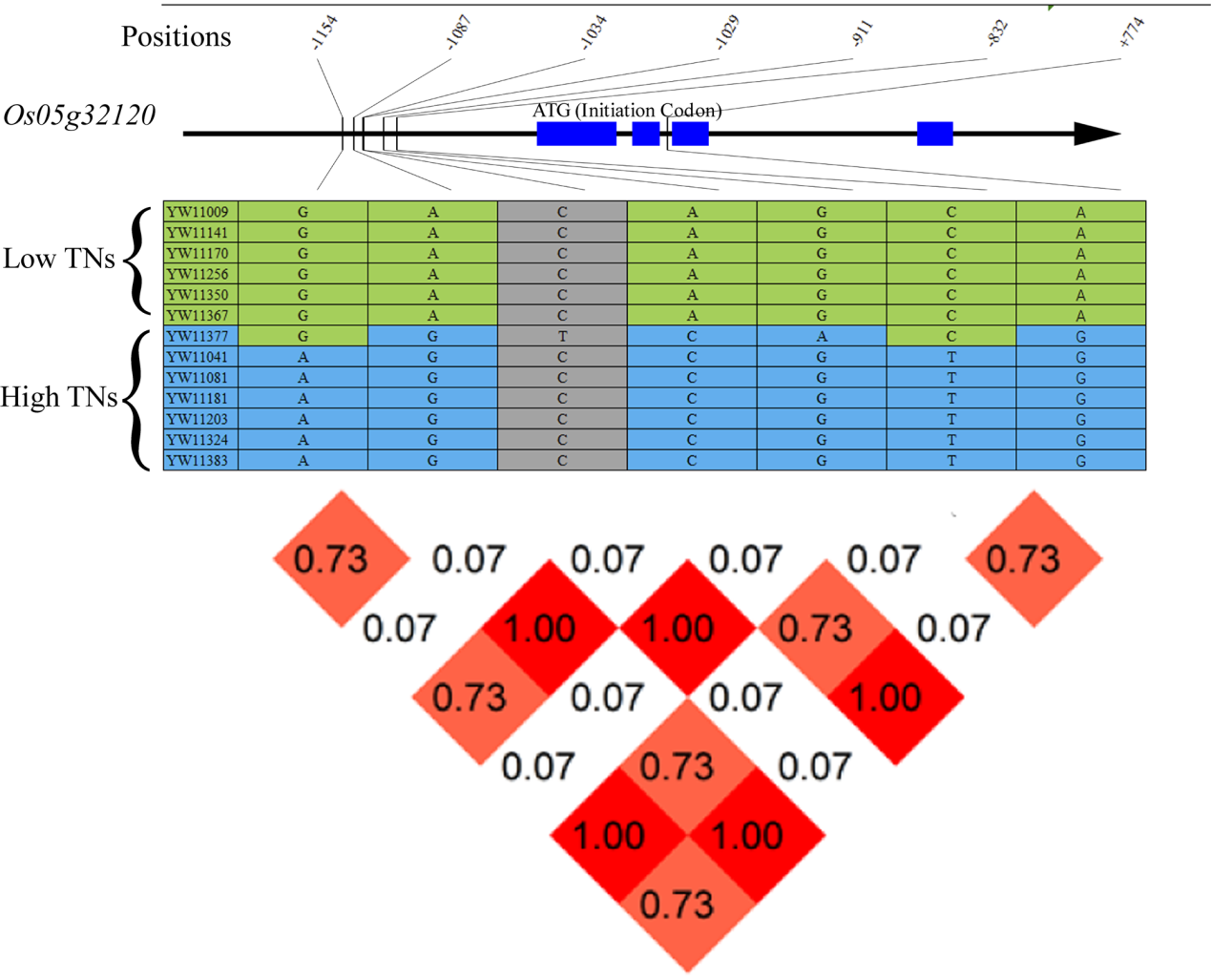


Figure S3. The haplotype analysis of the candidate gene *Os05g32120* in the accessions with extremely low and high TN phenotypes.


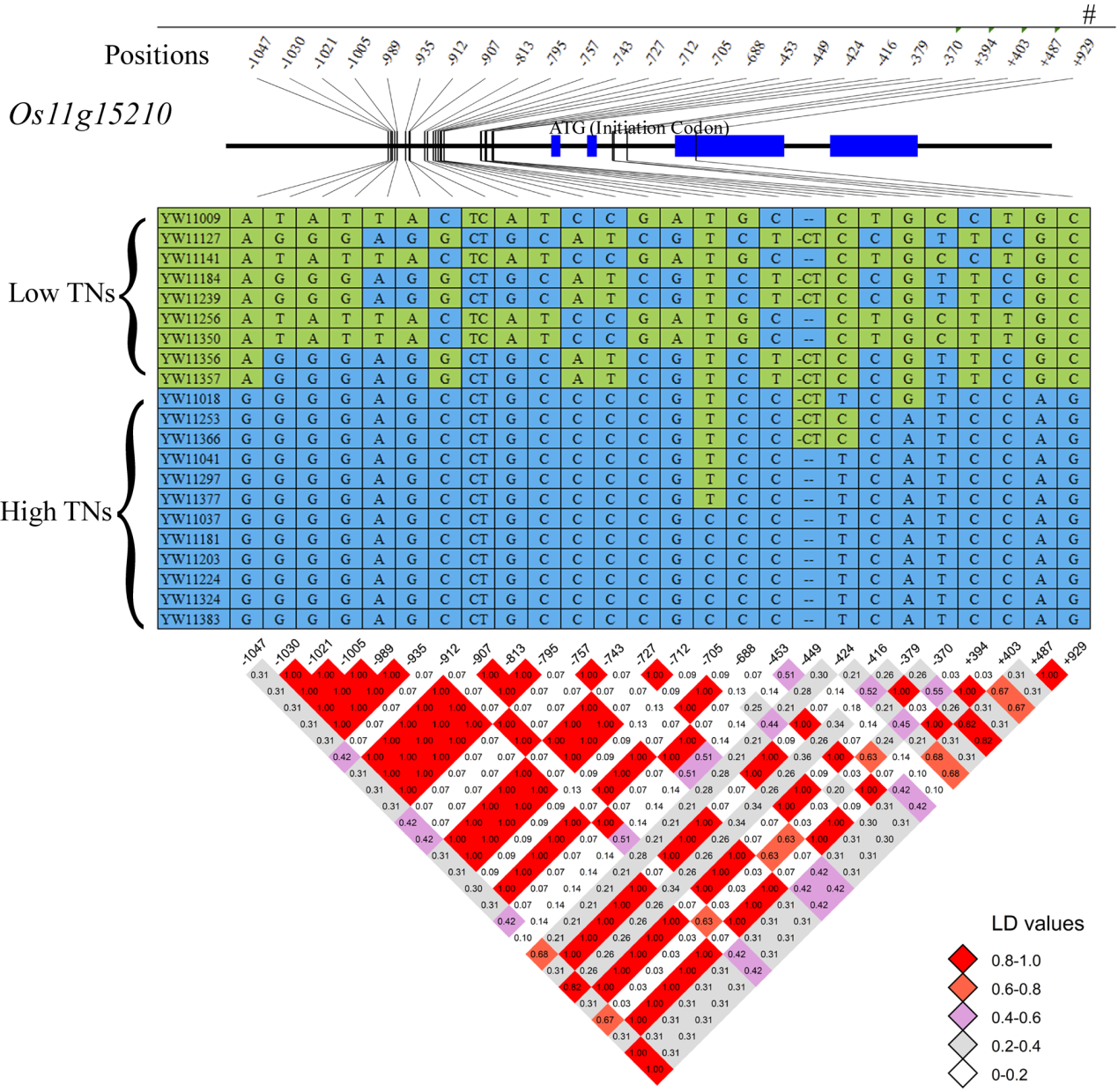


Figure S4. The haplotype analysis of the candidate gene *Os11g15210* in the accessions with extremely low and high TN phenotypes.
